# Progress of Surgery

**Published:** 1874-04

**Authors:** Jno. E. Owens

**Affiliations:** Lecturer on Surgery, Rush Medical College, Chicago


ARTICLE III.—*Progress of Surgery.* By JNO. E. OWENS, M.D., Lecturer on Surgery, Rush Medical College, Chicago.

1. *Ovariotomy.* By Mr. SPENCER WELLS. (A Biennial Retrospect of Medicine and Surgery for 1871-72. New Sydenham Society, 1873. British Medical Journal, Dec., 1872.)

2. *Ovariotomy on a Child.* By Mr. ALCOCK. (London Lancet, December, 1871.)

3. *Ligation of Pedicle in Ovariotomy.* By Dr. HAYES. (Dublin Quarterly Journal, Nov., 1871.)

4. *Ovariotomy on a Child.* By Dr. W. BARKER. (Philadelphia Medical Times, Nov. 1, 1871, American Journal of the Med. Sciences, Jan., 1872.)

5. *The Present State of our Knowledge of the Pathogenesis of Tumors.* By Prof. S. D. GROSS. (American Journal of the Med. Sciences, Jan., 1874.)

6. *Esmarch's Method of Preventing Hemorrhage During Operations.* (London Lancet, Oct. 11, 1873.)

7. *Growth of Cicatrices.* By Mr. W. ADAMS. (The Practitioner, London, Jan., 1874.)

8. *Treatment of Strangulated Hernia by Aspiration.* By Mr. DUBREUIL, (Medical Times and Gazette, Nov. 22, 1873. The Practitioner, London, Jan., 1874.)

1. Mr. Spencer Wells has lately ("Med.-Chir. Soc.," Nov. 1872) completed the analysis of 500 cases of ovariotomy performed by him. In 25 cases both ovaries were removed at one operation; and there were four cases where ovariotomy was performed twice on the same patient. The subsequent history of patients who recovered after removal of one ovary proved that they might menstruate regularly, and might bear children of both sexes, or twins; and that, after removal of both ovaries, they did not become excessively fat, nor lose their feminine appearance or sexual instinct.

Of 373 women who recovered, 36 who were unmarried at the time of the operation had married since. Of these, 15 had had one child, 6 two children, 3 three children, 3 four children, and 2 had had twins. Of 259 who were married when the operation was performed, many being beyond the age of child-bearing, 23 had had one or more children since; 17 had died of causes more or less directly connected, and 19 of causes not at all connected, with ovarian disease or the operation, at various periods from a few weeks to eight years after ovariotomy. Mr. Wells stated that unilocular cysts often disappeared after a single tapping. They were frequently not ovarian at all, but connected with the parovarium or broad ligament. His views as to early operations had become modified from those which he at first held. He had found that the results of operations on small tumors in healthy persons were not so favorable as in cases where the cysts were large, and the patients had become accustomed to disease. He did not think it right to remove small ovarian tumors, unless they caused great pain and inconvenience to the patient.

2. Mr. Alcock operated on a child three years old. The tumor was universally adherent. The patient died at the end of forty-eight hours.

3. Dr. Hayes recommends a method for securing the vessels of the pedicle (ovarian), which he calls "the subperitoneal."

The proceeding resembles the subcutaneous ligature of nævus. The pedicle is first compressed by a clamp, and a needle, armed with a stout catgut ligature, is passed beneath a good thickness of the serous surface of the pedicle, but superficial to the principal vessels. The needle being withdrawn at the side opposite the point of entrance, is again passed into the aperture of exit, and pushed between the vessels and peritoneal covering on one side of the vessels opposite its first passage, until it can be withdrawn through the opening made by its first entrance. The ends of the ligature are to be strongly tied and cut off short.

4. Dr. W. Barker has operated on a child aged six years and eight months. The case was one of dermoid cyst of the right ovary. The patient recovered.

5. Prof. Gross thus concludes a paper on "The Present State of our Knowledge of the Pathogenesis of Tumors:"

"All our views in regard to the pathogenesis of tumors are hypothetical, and rest on no solid foundation. No one has hitherto seen, for example, the cell of carcinoma originate from the stable connective-tissue corpuscle, epithelium, or the wandering cell. Conclusions are drawn from the representations afforded by dead tissues; and it is not probable that direct observations will ever be made on this point. Hence it is the duty of the impartial critic to examine the immense mass of enticing views that have been brought forward for the support of the various theories of the development of morbid growths, and adopt that which strikes him as being the least objectionable. Were I disposed to confine myself to a single hypothesis, I should advocate the doctrine that the wandering or mobile connective-tissue cell is directly transformed into the tissues which enter into the composition of the neoplasms; not by any independent action of its own, but by the law of analogy of formation. The adoption of such a theory is, however, scarcely warranted by the present unsettled state of our knowledge; so that I am led to sustain the more conservative views of many distinguished observers and experimenters, who teach that all the cells of the organism, whether fixed or mobile, epithelial or connective, investing or secreting, lend their aid in the development of tumors, but with the restriction, on my own part, that the migratory cell is principally concerned, as in the inflammatory process. In other words, the genesis of morbid growths is characterized by the hypernutrition and multiplication of the masses of protoplasm influenced by it, which are, as a rule, direct productions of the vascular and connective-tissue systems."

6. No ill effects of any kind have hitherto been observed (in London) from the use of this contrivance. Although the duration of the operations has varied from a few minutes up to half an hour, and even more, during the whole of which time the circulation has been completely arrested, no evidence has been afforded of the formation of emboli or thrombi in any cases. But it is one of the possible evils of the device that the prolonged pressure on the vessels and complete stoppage of circulation may, under certain conditions, lead to the formation of a clot, which, on the re-establishment of the circulation, may be carried along the vessels, and arrested in some part of their course, giving rise to circumscribed inflammation or even gangrene. There is also considerable danger in applying the bandage over parts which are inflamed and suppurating, especially if decomposition be going on, lest some of the clots which are found in the blood vessels of the affected parts be detached and forced into the blood current. For such cases it would be well to employ, in addition, a modification of the plan which has been practiced at Edinburgh for the last two or more years, and which consists in suspending the limb for some minutes before the operation, so that the blood may gravitate downward. Then the bandage may be applied at the proximal side of the diseased parts, thus avoiding all risks of septic poisoning or of embolism.

7. Mr. W. Adams, in a paper read before the Medical Society of London, demonstrates that scars made in children grow with the general growth of the body. He exhibited casts taken at different periods of life, in some of which a growth of as much as an inch had taken place in the course of six or seven years. After deep wounds, or when a portion of skin has been destroyed, the cicatrix appears to be persistent through life. Although all cicatrices, at the time of their formation, are much less than the wounds from which they result, still if the wounds should be made in early childhood, the resulting cicatrix will be, at the completion of growth, very much larger than the original wound; but cicatrices of wounds made after the completion of growth maintain through life the same proportions. With regard to the wearing out of scars, Mr. Adams thinks that those scars only wear out which result from superficial cuts which do not penetrate fairly through the deep layers of the skin into the subcutaneous fat.

8. Mr. Dubreuil has recently read a report or paper by M. Dunlafoy on the treatment of strangulated hernia by aspiration. In this he relates twenty-seven cases, showing the great utility of this procedure in many cases and its innocuity in all. It is true it implies that a diseased intestine may be returned, but the same remark applies to the taxis. M. Dubreuil, in a discussion which followed the reading of the paper, stated that he could not accept all the conclusions of the author. He did not admit that aspiratory puncture should be the first means resorted to, believing that moderate taxis, which is always inoffensive, should first be tried; but he did not hesitate to recognize that aspiration constitutes a real advance in the treatment of strangulated hernia. Both M. M. Verneuil and Trilat also admitted that in certain cases aspiration is very useful, as when the strangulated hernia is complicated by effusion into the sac, the withdrawal of this liquid by aspiration much facilitates exact diagnosis, and allows of a hernia being reduced which had previously resisted numerous efforts by the taxis. This can be brought to bear directly upon the intestine, which is no longer marked by the presence of the liquid. Aspiration thus at once becomes explorative and curative. M. Trilat took the same view of the operation, but M. Lee stated his opinion that all that is necessary may be effected by means of a trocar. M. Verneuil pointed out that the evacuation of the liquid by a fine trocar is a matter of difficulty, whilst it is easily accomplished by aspiration. M. Despres, the irreconcilable adversary of aspiration, predicted for it a similar failure. In his opinion there is no other treatment for strangulated hernia than operating.

